# Incorporating biological modeling into patient‐specific plan verification

**DOI:** 10.1002/acm2.12831

**Published:** 2020-02-26

**Authors:** Ara N. Alexandrian, Panayiotis Mavroidis, Ganesh Narayanasamy, Kristen A. McConnell, Christopher N. Kabat, Renil B. George, Dewayne L. Defoor, Neil Kirby, Nikos Papanikolaou, Sotirios Stathakis

**Affiliations:** ^1^ Department of Radiation Oncology University of Texas Health Sciences Center San Antonio TX USA; ^2^ Department of Radiation Oncology University of North Carolina Chapel Hill NC USA; ^3^ Department of Radiation Oncology University of Arkansas for Medical Sciences Little Rock AR USA

**Keywords:** IMRT QA, radiobiological QA, radiobiological verification, radiobiology

## Abstract

**Purpose:**

Dose–volume histogram (DVH) measurements have been integrated into commercially available quality assurance systems to provide a metric for evaluating accuracy of delivery in addition to gamma analysis. We hypothesize that tumor control probability and normal tissue complication probability calculations can provide additional insight beyond conventional dose delivery verification methods.

**Methods:**

A commercial quality assurance system was used to generate DVHs of treatment plan using the planning CT images and patient‐specific QA measurements on a phantom. Biological modeling was performed on the DVHs produced by both the treatment planning system and the quality assurance system.

**Results:**

The complication‐free tumor control probability, *P*_+_, has been calculated for previously treated intensity modulated radiotherapy (IMRT) patients with diseases in the following sites: brain (−3.9% ± 5.8%), head‐neck (+4.8% ± 8.5%), lung (+7.8% ± 1.3%), pelvis (+7.1% ± 12.1%), and prostate (+0.5% ± 3.6%).

**Conclusion:**

Dose measurements on a phantom can be used for pretreatment estimation of tumor control and normal tissue complication probabilities. Results in this study show how biological modeling can be used to provide additional insight about accuracy of delivery during pretreatment verification.

## 1 INTRODUCTION

Intensity‐modulated radiotherapy (IMRT) has improved modern radiation oncology.^1^, ^2^, ^3^, ^4^, ^5^ Compared to conventional beam delivery techniques, inverse planning results in unique fluence maps for each beam. Volumetric modulated arc therapy (VMAT) adds to the complexity with gantry rotation, dose rate variation, and motion of multi leaf collimators during treatment. Complexity demands that stringent patient specific quality assurance (QA) measures be implemented to ensure agreement between calculated treatment plans and delivered IMRT dose distributions. A widely adopted QA metric, called gamma index, was developed to evaluate and provide clinical confidence in IMRT treatment plans. The gamma index accounts for both differences in dose and geometry to quantify the agreement between calculated and measured dose.^6^, ^7^, ^8^, ^9^

The gamma index is used to provide confidence when evaluating accuracy of delivery; however, this analysis does not provide detailed dosimetric information about specific structures as well as hot or cold spots in the target.^10^, ^11^ Furthermore, studies have shown that gamma‐based analysis can be insensitive to detect errors or correlate dose errors in anatomic regions of interest.^12^, ^13^ Results derived from the usual individualized pretreatment QA tools have not been related with clinically relevant dosimetric errors on patient dose delivery. A more robust QA than the gamma index would be needed to quantify the clinical impact of dose measured prior to treatment in comparison to planned dose distribution, in addition to estimating the radiobiological implications of any dose differences.

A new approach for plan verification compares independently measured dose–volume histograms (DVHs) to that computed by a treatment planning system (TPS). There are commercially available solutions that incorporate dose measurements on phantom with the CT images of patient to compute pretreatment DVH. The capabilities of producing DVH of the Delta4DVH Anatomy 3D QA system (Scandidos, Uppsala), and both MapCHECK 2 and the ArcCHECK with 3DVH system (Sun Nuclear, Melbourne) have been evaluated in previous studies.^14^, ^15^, ^16^ New metrics for IMRT QA verification were explored in a study that utilized the COMPASS system (IBA Dosimetry, Bartlett, Tennessee) to incorporate pretreatment DVH into tumor control probability (TCP) and normal tissue complication probability (NTCP) models.^17^

TCP provides additional insight to plan quality as it is associated with the clinically observed tumor control rates. Similar association exists between NTCP and radiation‐induced toxicity to organs at risk (OAR). These radiobiological metrics offers accountability for the response of specific tissues to dose and dose per fraction, which is not considered in the gamma index.^18^ In previous studies, the complication‐free tumor control probability, *P*_+_, has demonstrated value in approximating complication rates of patients treated.^19^

The aim of this work is to demonstrate the value of incorporating *P*_+_ as a pretreatment verification metric for IMRT plans.

## 2 MATERIALS AND METHODS

### 2.1 Patient cohort

Fifty‐four previously treated VMAT patient plans were used in this study. Prescribed doses and fraction schedules were dependent on the treatment site; however, no patients in the cohort received stereotactic body radiotherapy or radiosurgery. The patients were treated for 5 different anatomical sites consisting of 10 brain, 10 head‐neck, 10 lung, 14 pelvis, and 10 prostate patients. There was a variation in the dose, number of fractions, and modalities incorporated in the treatment plans (Table 1). No patients with extreme circumstances such as prosthetic implants or unusual physiological conditions were included in the study. All structures analyzed in this study are summarized in Table 1.

**Table 1:** Summary of all structures used in TCP/NTCP comparison.

Cohort	Dose range [Gy]	Fractions	Modality	Structures for TCP/NTCP comparison
Brain	50.40‐60.00	28‐30	6X	Brain, brainstem, chiasm, Rt. optic nerve, Lt. optic nerve, PTV
Head‐neck	30.00‐69.96	5‐35	6X	Rt. parotid, Lt. parotid, mandible, Rt. brachial plexus, Lt. brachial plexus, PTV
Lung	30.00‐60.00	3‐30	6X, 10X	Esophagus, heart, lung, PTV
Pelvis	34.20‐79.20	11‐28	6X, 10X	Bladder, rectum, sigmoid, bowel, penile bulb, PTV
Prostate	45.00‐70.20	25‐30	10X	Bladder, rectum, sigmoid, penile bulb, PTV

High‐risk and low‐risk PTV were used for head‐neck analysis.

Patient plans were planned in Pinnacle^3^ TPS (Philips, Koninklijke, Netherlands). The plans were delivered into the Octavius 4D phantom (PTW, Freiburg, Germany), and resulting measurements were compared to the planned dose using conventional gamma analysis. Two criteria used to test the data were dose difference/distance‐to‐agreement (DTA) of 3%/3 mm and 2%/2 mm normalized to 90% of max dose. The institutional criteria for all these plans were 90% pass rate for all pixels at 3%/3 mm normalized to 90% of max dose. 2%/2 mm criteria results are included to examine if simply tightening gamma criteria is indicative of accuracy of delivery.

**Figure 1:**
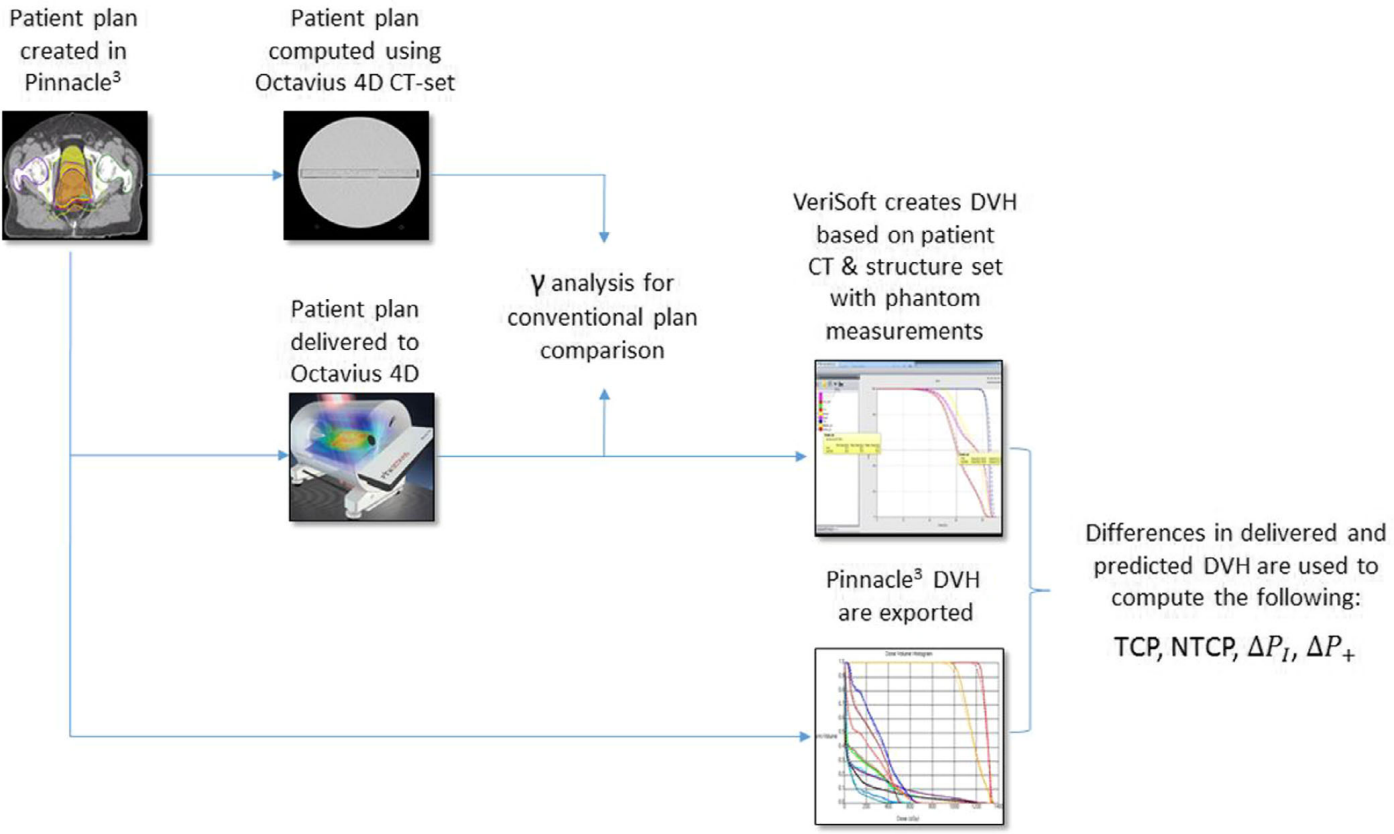
Steps of this study are shown in this workflow diagram.

Phantom measurements were used with the VeriSoft software ver 7.0 (PTW, Freiburg, Germany) to construct delivered DVH by scaling the measurements onto the patient CT set. The patient's DVHs that were originally computed in Pinnacle^3^ are then exported to perform radiobiological calculations (details below). The workflow is outlined in Fig. 1.

### 2.2 Systems used

Plans were delivered using the same Novalis Tx LINAC (Varian Medical Systems, Palo Alto, California) to mitigate differences that could occur from plan delivery with different LINACs due to variations in treatment planning beam models or configurations.

Pinnacle^3^ TPS was used for calculating patient plans in addition to creating plans to be delivered to the phantom under the same conditions. A 2.5‐mm^3^ voxel grid resolution was used for TPS calculations. After completing calculation, DICOM (DCM) files consisting of plans, structures, dose, and CT image sets were exported to be used with VeriSoft DVH calculations later. Phantom DCM were created by importing plan configurations onto the CT study of an Octavius 4D phantom set that was previously acquired. The phantom DCM consisting of the plan's dose was exported to be used in gamma analysis.

An Octavius 1500D detector array was used with the four‐dimensional (4D) phantom. The phantom is cylindrical and is designed to rotate around its base in synchrony with the gantry rotation. An inclinometer, attached to the base of the gantry, sends information about its position to steer the rotation of the phantom's cylindrical body. There is a 200 ms measuring interval to ensure good correlation between dose data and gantry angles. This measuring interval combined with an inherent uncertainty of the gantry angle for the three‐dimensional (3D) dose reconstruction algorithm leads to a total uncertainty of ±1.2° for gantry speeds up to 360° per minute.^20^ The Octavius 1500 consists of 1405 0.06 cm^3^ vented cubic ion chambers (0.44 cm^3^ × 0.44 cm^3^ × 0.30 cm^3^) mounted beneath a 0.5 cm polystyrene build‐up layer. The ion chambers cover a 27 cm^3^ × 27 cm^3^ active area in a checkerboard pattern and are spread spatially with a center‐to‐center distance of 0.707 cm. Dose measurements were recorded and processed using VeriSoft. Once the measurements are collected, VeriSoft can reconstruct a 3D dose volume for comparison with the computed dose distribution in the phantom by the TPS. The reconstruction algorithm is based on percent depth dose (PDD) curves for different field sizes. First, the PDDs measured in water are converted to PDDs in the Octavius 4D phantom using the known relation of electron densities of water and phantom material. The PDD data are established at the time of the initial setup of the VeriSoft application during commissioning of the Octavius 4D system. PDD data for various field sizes ranging from 4 cm^2^ × 4 cm^2^ to 26 cm^2^ × 26 cm^2^ are entered into the system as part of the commissioning process.

At each gantry angle (parametrized as time), each detector of the panel measures a dose. For every gantry angle, the detector array measures a dose plane that is perpendicular to the incident beam. A ray through each detector of the panel is back‐projected to the source and the field size is determined through the irradiated detectors. During a measurement session, the effective field size is determined in real time by the software by integrating the position of the detectors that received radiation above a certain threshold. All dose points (measured and extrapolated at a given gantry angle) are summed over all gantry angles of the delivery to create a 3D dose distribution. Using the PDD appropriate to field sizes, the dose values along the ray lines that connect the irradiated detectors and the beam focus were reconstructed.^20^

**Figure 2:**
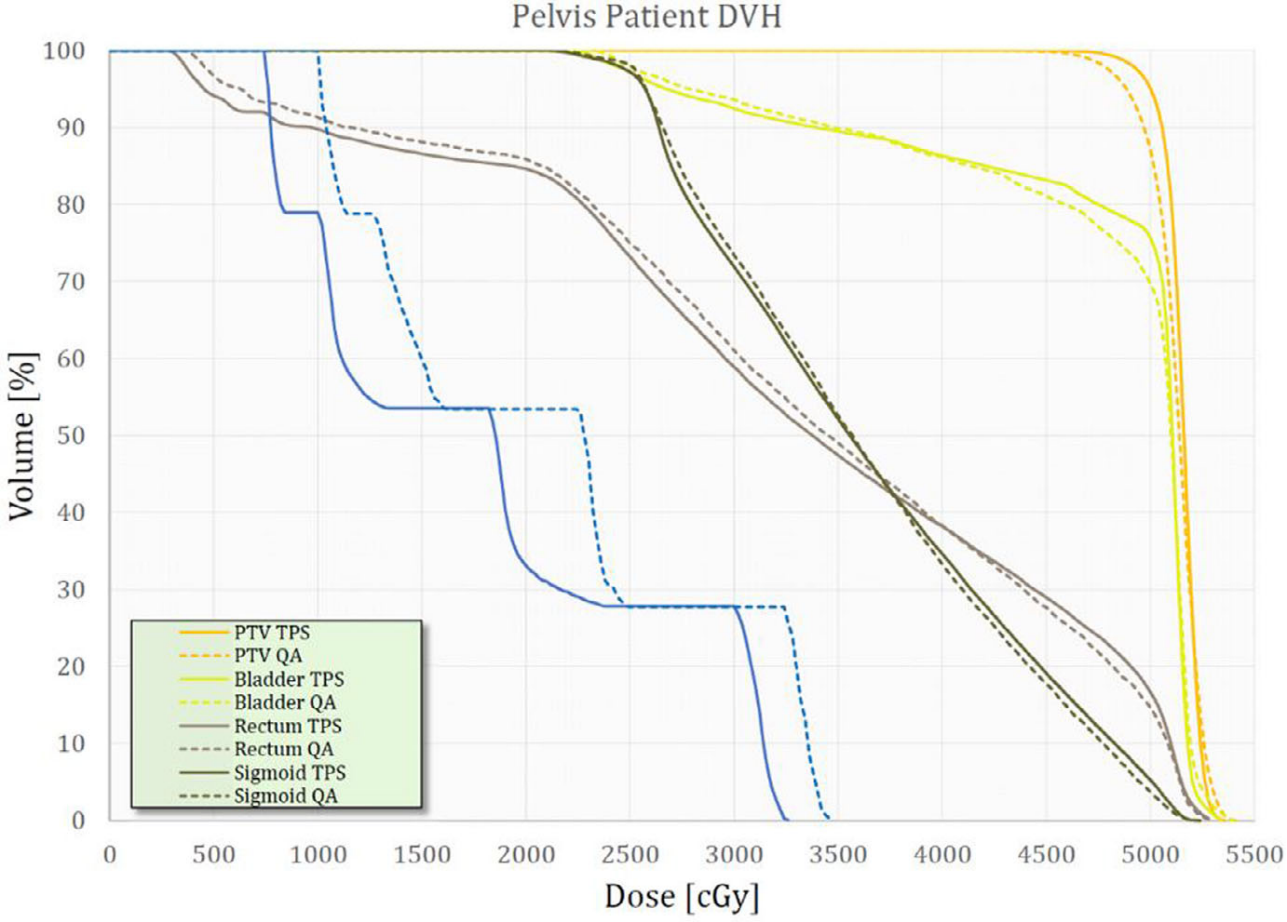
An example of a dose–volume histogram (DVH) set produced by Pinnacle3 and VeriSoft for a pelvis patient. The solid lines correspond to DVH from Pinnacle3 and the dashed lines correspond to DVH from VeriSoft.

VeriSoft allows the user to perform slice‐by‐slice two‐dimensional (2D) and 3D gamma index calculation, slice‐by‐slice comparison of the measured and computed dose distributions and dose profiles comparison. A volumetric 3D gamma index can be calculated for the entire 3D dose distribution, comparing the TPS calculations and the reconstructed 3D dose from the measurements. VeriSoft utilizes a pencil beam algorithm for computing dose and path length scaling to deal with inhomogeneity. This is less rigorous than the collapsed cone algorithm used in Pinnacle^3^ where kernel tilting is utilized to account for tissue inhomogeneity; however, it provides a tradeoff advantage in calculation speed which is beneficial during clinical time constraints.^21^, ^22^ VeriSoft imports patient structure sets from the initial TPS structure delineations and utilizes the 4D dose distributions from measurements to reconstruct patient DVH data. The version of the software (version 7.0) used in this study is unable to produce information for structures that do not entirely fit on the PTW 1500 matrix array surface area during dose acquisition. Figure 2 shows DVH produced with Pinnacle^3^ overlaid with VeriSoft's DVH for the patient.

### 2.3 TCP/NTCP modeling

A radiobiological evaluation was performed between the Pinnacle^3^ computed plans and the ones calculated from the phantom measurements. The DVH of organs represented in the plan pairs were used for estimation of radiobiological metrics. The formula that is used to calculate the response of each voxel or bin in a DVH for tumors and normal tissues is based on the Poisson model^23^, ^24^, ^25^:

(1) $$P\left(D\right)=\exp\left(e^{e\gamma -\left(D_{2\;\text{Gy}}/D_{50}\right)\left(e\gamma -\ln\ln2\right)}\right),$$

where *P(D)* is the probability of response of a voxel irradiated by uniform irradiation of dose *D*, *D*_50_ is the dose that induces response to 50% of the patients, and γ is the maximum normalized dose‐response gradient. In Eq. (1), the fractionation correction of dose is handled by using the quantity *D*_2 Gy_, which is the equivalent dose at 2 Gy per fraction.^26^ The *D*_2Gy_ is calculated by the following expression:

(2) $$EQD_{2\;\text{Gy}}=\frac{D_{x\;\text{Gy}}\left(x+\frac{\alpha }{\beta }\right)}{\left(2+\frac{\alpha }{\beta }\right)},$$

where *D_x_*_ Gy_ is the total dose when *x Gy* is the dose per fraction. To estimate normal tissue complications (NTCP) from nonuniform dose distributions, the relative seriality model was used:

(3) $$\text{NTCP}={\left[1-\prod_{i=1}^{M}{\left(1-\exp{\left[-e^{e\gamma -\left(EQD_{2\;\text{Gy}}^{i}/D_{50}\right)\left(e\gamma -\ln\ln2\right)}\right]}^{s}\right)}^{\Delta v_{i}}\right]}^{1/s}.$$

The overall probability of injury, *P*_I_, for several OARs is expressed by the following equation^24^, ^25^:

(4) $$P_{I}=1-\prod_{j=1}^{N_{\text{organs}}}\left(1-{\text{NTCP}}_{j}\right),$$

where *N*_organs_ is the total number of vital OARs, and NTCP*_j_* is the response probability of the organ *j* having the reference volume and been irradiated by a dose *D_i_* as given by Eq. (1). Furthermore, Δ*v_i_* is the fractional subvolumes of the organ being irradiated, *M* is the total number of voxels or subvolumes in the considered organ, and *s* is relative seriality parameter of that organ. In tumors, for estimating tumor control probability from nonuniform dose distributions, the following model was used:

(5) $$\text{TCP}=\prod_{i=1}^{M}{\left(\exp\left[-e^{e\gamma -\left(EQD_{2\;\text{Gy}}^{i}/D_{50}\right)\left(e\gamma -\ln\ln2\right)}\right]\right)}^{\Delta v_{i}}.$$

The overall probability of benefit, *P*_B_, can be quantified by the following expression:

(6) $$P_{B}=\prod_{j=1}^{N_{\text{tumors}}}\left({\text{TCP}}_{j}\right),$$

where *N*_tumors_ is the total number of tumors or targets involved in the clinical case. The effectiveness of different treatment plans were evaluated by the radiobiological concept of complication‐free tumor control probability, *P*_+_, which represents the probability of achieving tumor control without causing damage to normal tissues.^27^, ^28^

(4) $$P_{+}=P_{B}-P_{B\cap I}\approx P_{B}-P_{I}.$$

The radiobiological analysis was based on the DVH from Pinnacle^3^ and phantom measurements using the VeriSoft software. The corresponding TCP, NTCP, *P*_I_, and *P*_+_ values were calculated. The difference in these values was obtained to estimate the expected clinical impact of the differences obtained by the dosimetric analysis. A detailed presentation of the software that was used for the radiobiological analysis can be found in the work by Su et al.^29^ Resulting TCP/NTCP calculated from doses reported by Pinnacle^3^ were used as reference values when comparing to TCP/NTCP calculations from doses reported by VeriSoft. Tables 2, 3, 4, 5 report the summary of the model parameter values used for the examined cancer cases.^30^, ^31^, ^32^, ^33^ D50 is the dose that is associated with the 50% response rate, γ is the maximum normalized value of the dose–response gradient, and *s* is the relative seriality parameter.^18^, ^19^, ^34^, ^35^

**Table 2:** Summary of the model parameter values for the brain group.

Brain group					
Organs	D_50_ (Gy)	γ	α	α/β	Endpoint
PTV	55.0	2.5	Na	10.0	Control
Brain	60.0	2.6	0.64	3.0	Necrosis, infarction
Brainstem	65.1	2.4	1.0	3.0	Necrosis, infarction
Chiasm/optic nerve	65.0	2.3	1.0	3.0	Blindness
Spinal cord	57.0	6.7	1.0	3.0	Myelopathy

**Table 3:** Summary of the model parameter values for head‐neck group.

Head‐Neck group					
Organs	D_50_ (Gy)	γ	α	α/β	Endpoint
PTV7000	51.0	7.5	Na	10.0	Control
PTV5400	44.0	4.0	Na	10.0	Control
Parotid gland	46.0	1.8	0.01	3.0	Xerostomia
Spinal cord	57.0	6.7	1.0	3.0	Myelopathy
Mandible	70.3	3.8	1.0	3.0	Marked limitation of joint function
Brachial Plexus	75.1	2.8	8.4	3.0	Nerve damage

**Table 4:** Summary of the model parameter values for the lung group.

Lung group					
Organs	D_50_ (Gy)	γ	α	α/β	Endpoint
PTV	49.2	1.0	Na	10.0	Control
Esophagus	68.0	2.8	3.4	3.0	Clinical stricture/perforation
Heart	70.7	0.96	1.0	3.0	Cardiac mortality
Lung	30.1	0.97	0.01	3.0	Radiation pneumonitis
Spinal cord	57.0	6.7	1.0	3.0	Myelopathy

**Table 5:** Summary of the model parameter values for the pelvis and prostate groups.

Pelvis and prostate group					
Organs	D_50_ (Gy)	γ	α	α/β	Endpoint
PTV7920 (prostate)	63.0	5.0	Na	3.0	Control
PTV6000 (pelvis)	55.0	3.0	Na	3.0	Control
Bladder	80.0	3.0	0.3	3.0	Symptomatic contracture
Rectum	80.0	2.2	0.7	3.0	Proctitis, necrosis, fistula, stenosis
Sigmoid	80.0	2.2	0.7	3.0	Ulceration
Bowel	60.0	2.1	0.14	3.0	Stenosis
Penile Bulb	70.0	2.5	0.7	3.0	Erectile Dysfunction
Femur head	65.0	2.7	1.0	3.0	Necrosis

## 3 RESULTS

### 3.1 Gamma analysis

Table 6 summarizes the resulting values for gamma analysis of all cohorts. ${\underline{\Gamma }}_{3D}$ is the average 3D gamma score, $\sigma_{{\underline{\Gamma }}_{3D}}$ is the uncertainty in gamma 3D scores, $\mu_{\text{arith}}$ is the arithmetic mean, $\sigma_{\mu_{\text{arith}}}$ is the uncertainty of the arithmetic mean, $\mu_{\text{med}}$ is the mean of the medians, and $\sigma_{\mu_{\text{med}}}$ is the uncertainty of the mean of medians. The column labeled ‘Range’ shows the minimum and maximum value of the gammas in the corresponding cohort.

**Table 6:** Resulting values for gamma analysis of all cohorts.

Site	Criteria	ϒ3D_average_ (%)	Range (%)	σ_ϒ3D_ (%)	μ‐arithmetic	σ_μ‐arithmetic_	μ_median_	σ_μ‐median_
Brain	3%/3 mm	96.8	94.2‐99.5	1.6	0.64	0.04	0.69	0.03
	2%/2 mm	85.9	80.4‐89.8	3.5	0.81	0.08	0.76	0.04
H&N	3%/3 mm	92.8	88.8‐95.8	2.3	0.71	0.06	0.73	0.03
	2%/2 mm	78.0	72.0‐83.1	3.3	1.01	0.15	0.82	0.03
Lung	3%/3 mm	93.2	89.1‐96.1	2.5	0.72	0.05	0.74	0.03
	2%/2 mm	77.0	71.2‐81.0	3.9	1.03	0.17	0.84	0.03
Prostate	3%/3 mm	95.9	91.9‐97.9	2.3	0.64	0.05	0.69	0.04
	2%/2 mm	85.3	76.3‐90.0	4.8	0.82	0.09	0.77	0.05
Pelvis	3%/3 mm	96.5	94.9‐97.4	0.9	0.60	0.04	0.78	0.73
	2%/2 mm	86.8	81.8‐89.4	2.6	0.78	0.05	0.73	0.04

**Figure 3:**
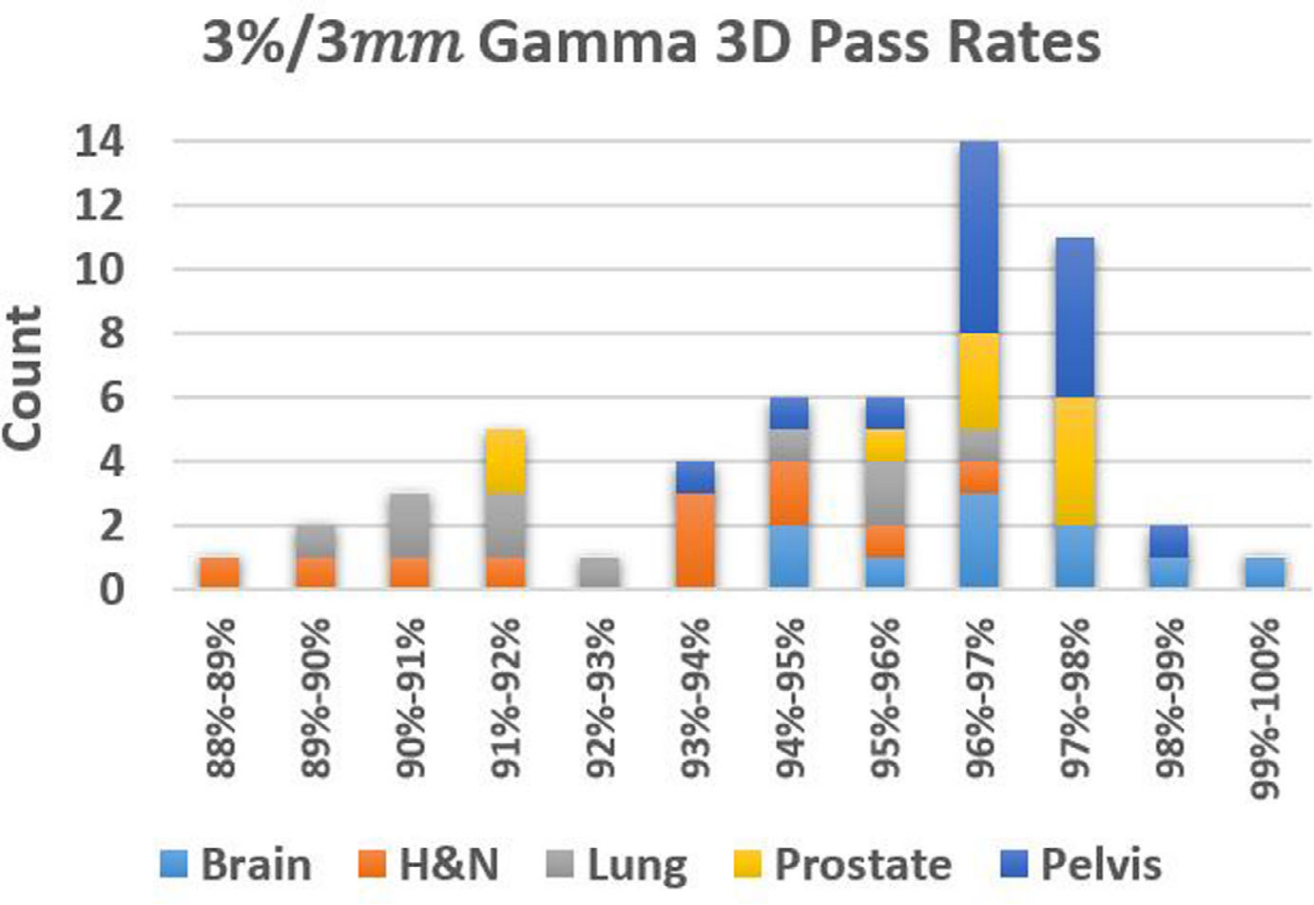
Histogram of Gamma 3D scores for all cohorts at 3%/3 mm tolerance.

**Figure 4:**
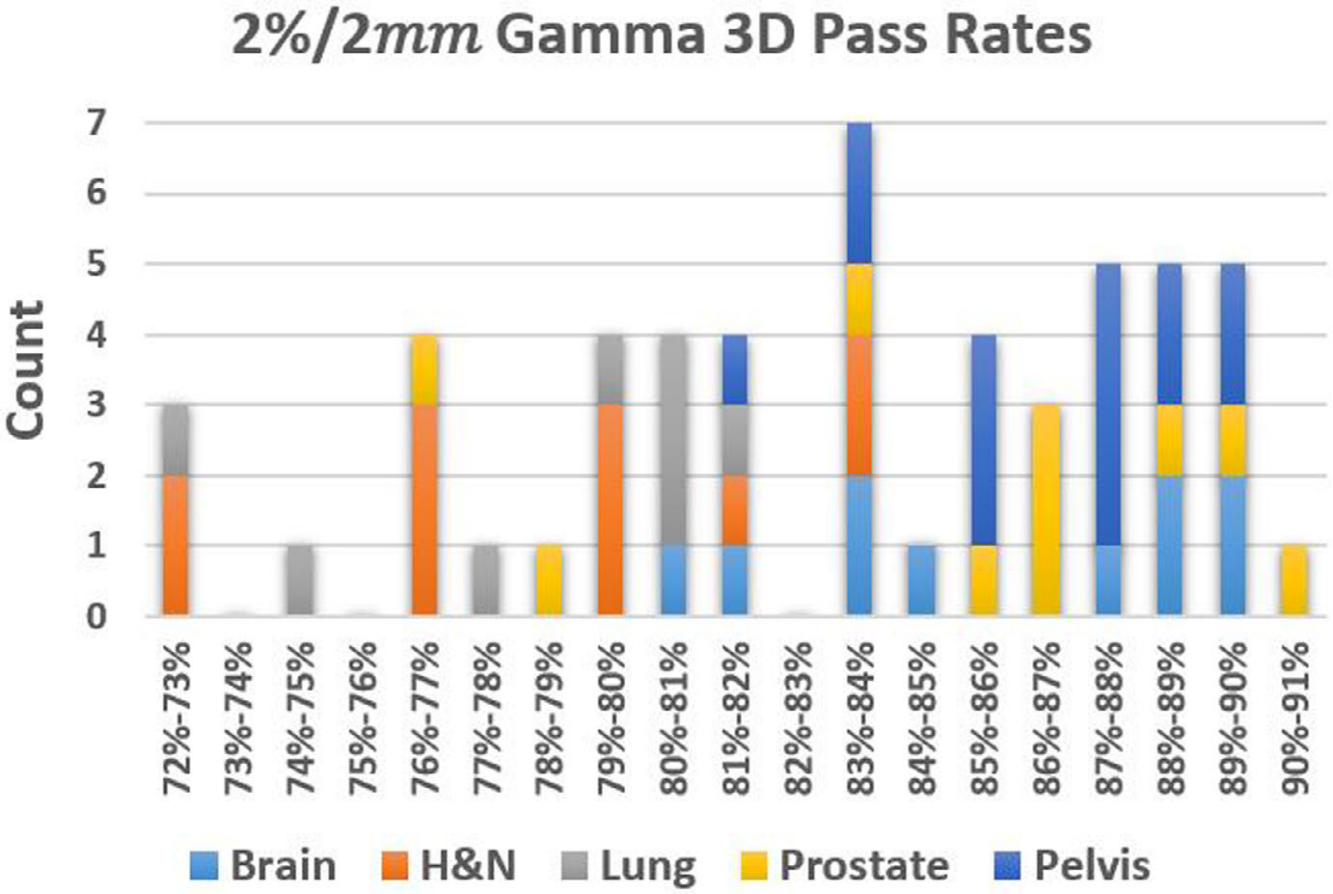
Histogram of Gamma 3D scores for all cohorts at 2%/2 mm tolerance.

A histogram of values for all the cohorts is shown in Figs. 3 and 4 to visualize the data for both 3%/3 mm and 2%/2 mm criteria.

### 3.2 TCP/NTCP results

Table 7 shows a comparison of mean ± standard deviation (stdev) of *P*_+_ values reconstructed using Verisoft and that computed in Pinnacle^3^. All TCP and NTCP values were estimated and individual *P*_+_ results can be found in Appendix A in Tables A1, A2, A3, A4, A5. A positive Δ*P*_+_ value means that the delivered plan results in higher complication‐free tumor control than the TPS computed plan data which is desirable. In contrast, negative Δ*P*_+_ values mean that the delivered plan results in lower complication‐free tumor control which indicates the delivered plan has poorer complication‐free tumor control than the TPS computed plan. Comparing the measured and computed dose distributions, 3D gamma values estimated using both 3%/3 mm and 2%/2 mm criteria are tabulated in Table 7.

**Table 7:** Mean ± standard deviation of *P*_+_ (Verisoft reconstructed), P__+_ (Pinnacle computed) data and their differences are tabulated along with gamma pass rates (3%/3 mm; and 2%/2 mm) for the 5 treatment sites.

Site	*P*_+_ (Verisoft)	*P*_+_ (Pinnacle3 TPS)	Δ*P*_+_	3D gamma value (3%/3 mm)	3D Gamma value (2%/2 mm)
Brain	57.4 ± 6.8	61.3 ± 11.1	−3.9 ± 5.8	96.8 ± 2.9	85.9 ± 5.6
H&N	44.3 ± 38.9	62.6 ± 31.5	−18.2 ± 43.6	92.8 ± 2.1	78.0 ± 3.5
Lung	83.5 ± 16.3	75.7 ± 19.4	7.8 ± 18.3	93.2 ± 2.5	77.0 ± 4.2
Prostate	60.1 ± 25.3	53.0 ± 33.4	7.1 ± 12.1	95.9 ± 1.2	85.3 ± 2.6
Pelvis	67.1 ± 25.3	66.3 ± 27.5	0.8 ± 3.6	96.5 ± 2.3	86.8 ± 4.8

## 4 DISCUSSION

Gamma analysis performed on the five different treatment sites resulted in an average 3D gamma index of 95 ± 2% with 3%/3 mm tolerance and 82 ± 4% with 2%/2 mm tolerance. All the plans in this study except three passed our institutions evaluation criteria when using conventional gamma analysis. At 88.8%, 89.1%, and 89.4%, it is plausible that these were challenging cases and were accepted slightly below the clinical threshold.

**Figure 5:**
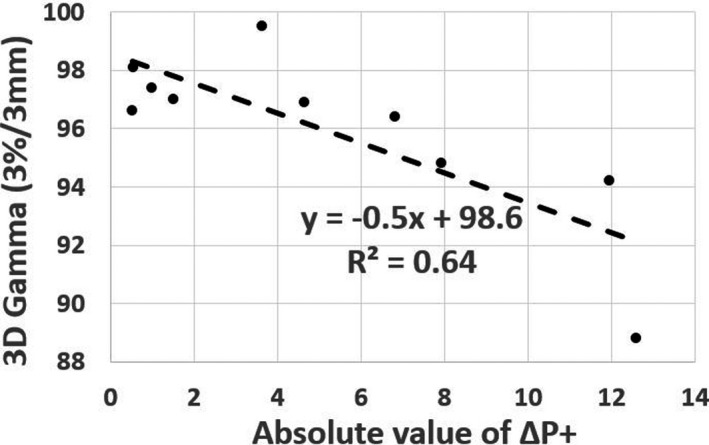
3D Gamma passing rates (3%/3 mm criteria) of brain cohort vs absolute value of Δ*P*_+_.

It is pertinent to state here that while the *P*_+_ values are based on the DVHs of target(s) and OARs, the gamma passing rates are determined across the dose grid volume on the entire CT dataset of the patient. It is natural to expect that high compliance among calculated and reconstructed DVHs (i.e., Δ*P*_+_ ≈ 0) occur in regions where gamma passing rates are high (γ ≈ 100%). Figure 5 demonstrates the correlation between 3D gamma value (using 3%/3 mm criteria) and the absolute value of Δ*P*_+_ on the brain cohort with a Pearson correlation coefficient, R^2^ = 0.64. Alternatively, significant differences between the measured and TPS computed doses can have big clinical impact if they coincide geometrically with critical structures that include targets, OARs. This exemplifies that gamma passing rates estimated for the entire volume does not provide region‐specific information of where a failure occurs, at which dose level or magnitude of dose error, and how clinically significant it could be.

Patients 5 and 6 in the brain cohort as tabulated in Table A1 in Appendix A had 94.8% and 96.4% gamma pass rates with 3%/3 mm tolerance, respectively. The calculated Δ*P*_+_ of the two plans was −7.94% and −6.82%, respectively, despite having clinically acceptable gamma pass rates. From Table A1, we can see that the measured dose predicts a reduction of TCP (PTV coverage) compared to the plan, ultimately resulting in a large negative difference.

Large reported differences in *P*_I_ and *P*_+_ values between the computed and delivered doses are observed in the head‐neck patients as tabulated in Table A2. It has been shown that dosimetric discrepancy of a few percent can have effects on TCP and NTCP.^36^, ^37^, ^38^, ^39^, ^40^, ^41^ This is also reflected in the low gamma passing rates of head‐neck plans. This may be attributed to poor spatial resolution of measurement against the computed dose in regions of steep dose falloff often witnessed in highly modulated head‐neck plans.

Patients 6 and 9 in the pelvis cohort resulted in Δ*P*_+_ values of +15.11% and +12.35%, respectively. This is due to the lower NTCP of the delivered DVHs compared to the computed DVHs. The TCP calculated from the delivered DVHs was lower than that from TPS computed DVHs resulting in a positive Δ*P*_+_ value (see Table A4). Similarly, the delivered dose to patient eight in the prostate cohort in Table A5 predicted lower TCP, resulting in a negative Δ*P*_+_ of −6.31%.

Agreement between measured and planned dose can be attributed to spatial resolution of detector panel, accuracy of Verisoft commissioning, dosimetric reconstruction accuracy, complexity of the treatment plan, etc. Large observed differences of TCP values should be investigated further by restricting cohorts to patients of identical pathology. For instance, forming a cohort of plans that only include targets that are superficial or deep seated can be considered in a future work.

There are precedence available in the literature. In a 2017 study on patient‐specific QA of IMRT plans, 2D gamma analysis alone could not assure radiobiological equivalence between planned and delivered dose.^42^ The authors concluded that radiobiological analysis in addition to physical dose comparison may provide adequate patient‐specific QA of IMRT plans. The variation in radiobiological metrics due to patient setup errors were simulated in a prostate study by Park et al.^43^ A radiobiological model‐based bioanatomical QA using TCP and NTCP provides feedback that cannot be evaluated by physical QA alone. In another study, Zhen et al studied the change in DVH, TCP, and NTCP metrics by intentional introduction of multileaf collimator (MLC) errors in 40 IMRT plans.^44^ By showing TCP and NTCP as both sensitive and specific metrics, the study concludes with a possibility of using changes in TCP (ΔTCP) and NTCP (ΔNTCP) as alternate QA metrics.

In an assessment study on the usefulness of biological metrics in patient‐specific QA, prostate VMAT plans of American Association of Physicists in Medicine (AAPM) Task group report 166 test cases were analyzed.^45^ In this analysis of two 3D verification systems, radiobiological parameters were incorporated into the individualized QA providing information complimentary to DVH metrics. Finally, Sumida et al's radiobiological gamma distribution was useful in identification of areas where dose is radio‐biologically different, not just physically different.^46^ The radiobiological gamma index (RGI) facilitates physician's understanding of the dose distribution from a clinical perspective. Our study results do not necessarily agree with Sumida et al that could be attributed to differences in device type, geometry, measurement and analysis techniques. While we used Octavius 1500D detector array to do cumulative dose analysis, Sumida et al had used per‐beam analysis on a MapCheck device. While the low doses from cumulative analysis could add up to be detectable by the device, the per‐beam analysis suppresses low dose for the individual beam. While Octavius 1500D array rotates with the gantry, MapCheck measurements are insensitive to gantry rotation due to *on‐faus* dose delivery. In addition, Sumida et al had used Niemierko's model to calculate TCP and NTCP, which was not utilized in this study.

TCP and NTCP models are based on some assumptions since they cannot account for all the involved biological and clinical mechanisms. For example, there are other factors such as chemotherapy, comorbidities that may impact dose response and consequently the determination of the model parameters of the different tumors and tissues. Additionally, there are uncertainties imposed by inaccuracies in patient imaging, treatment planning, patient setup and treatment delivery during radiotherapy. Consequently, the determined model parameters and the corresponding dose–response curves are characterized by confidence intervals. The results of this study depend on the accuracy of the radiobiological models and the parameters that describe the dose‐response relations of the different tumors and normal tissues. Most of those parameters have been derived from recently published clinical studies, where the confidence intervals have been reduced. For certain tissues, larger uncertainties are involved in the determination of their parameters. In those cases, the calculated TCP or NTCP values should be not seen as prediction of patient outcome but to assess uncertainty of the delivered dose.

## 5 CONCLUSION

Currently available commercial QA systems allow for users to compute DVHs using phantom measured dose, enabling more insightful QA methods to develop. This study demonstrated the ability to integrate TCP/NTCP modeling with cumulative DVHs produced with phantom measured dose. By incorporating TCP/NTCP models, the risk of a plan to result in injury by deviations in measured dose to normal tissues or tumor coverage can be assessed as a QA metric. Although there are no clinical requirements or literature‐based thresholds for maximum deviation allowed between TCP/NTCP values calculated for structures, guidelines can be determined for establishing thresholds. In certain cases, this can be useful for critical structures in patients that have pre‐existing pathologies to a structure or are approaching dose limits due to previous treatments. In cases showing considerable reductions in tumor control and increases in normal tissue complications, a replanning could be proposed giving more emphasis in the robustness of the plan especially regarding the degree of modulation used and the steepness of dose falloff around the target.

## CONFLICT OF INTEREST

No conflicts of interest.

## Appendix A TCP–NTCP results

**Table A1:** TCP/NTCP calculation results for brain patients.

Patient	TCP (%)	NTCP (%)					*P*_I_ (%)	*P*_+_ (%)
	PTV	Brain	Brainstem	Chiasm	RON	LON		
*Brain: Pinnacle*								
1	79.6	0.1	0.6	1.0	0.6	0.0	2.2	77.8
2	69.2	0.3	1.4	2.2	0.0	7.8	11.4	61.4
3	69.6	6.7	1.1	0.0	0.0	0.0	7.8	64.2
4	67.2	1.5	0.0	0.0	0.0	0.0	1.5	66.2
5	74.8	7.9	0.0	0.0	0.0	0.0	7.9	68.9
6	74.7	—	0.0	0.0	0.0	—	0.0	74.7
7	73.9	21.7	0.0	7.9	3.7	1.9	31.9	50.4
8	74.8	11.5	0.5	3.9	11.5	0.0	25.1	56.0
9	74.6	6.5	28.0	5.4	0.0	0.0	36.3	47.5
10	74.7	0.2	0.0	27.8	3.4	11.5	38.3	46.0
*Brain: VeriSoft*								
1	65.5	0.0	0.0	0.4	0.1	0.0	0.5	65.2
2	63.8	0.2	0.6	0.3	0.0	2.0	2.9	61.9
3	62.3	4.0	0.4	0.0	0.0	0.0	4.5	59.5
4	54.7	0.9	0.0	0.0	0.0	0.0	0.9	54.2
5	63.6	4.2	0.0	0.0	0.0	0.0	4.2	60.9
6	67.9	—	0.0	0.0	0.0	—	0.0	67.9
7	69.0	17.8	0.0	8.6	3.3	1.7	28.5	49.4
8	67.8	8.0	0.4	2.2	6.8	0.0	16.5	56.6
9	63.8	3.5	19.2	1.5	0.0	0.0	23.2	49.0
10	75.2	0.1	0.0	22.7	2.5	12.2	33.9	49.7

LON, left optic nerve; RON, right optic nerve.

**Table A2:** TCP/NTCP calculation results for head‐neck patients.

Patient	TCP (%)		NTCP (%)					*P*_I_ (%)	*P*_+_ (%)
	PTV‐HR	PTV‐SR	Parotid‐R	Parotid‐L	Mandible	Brach‐Plex‐R	Brach‐Plex‐L		
*Head‐Neck: Pinnacle*									
1	95.9	98.4	0.0	0.0	0.0	0.0	0.0	0.0	94.4
2	100.0	98.9	1.3	24.3	25.9	29.3	35.5	74.8	25.0
3	95.0	—	—	0.0	0.2	—	—	0.2	94.7
4	100.0	98.7	0.0	0.0	20.8	0.0	2.6	22.8	76.2
5	—	97.7	0.0	0.0	1.0	55.6	1.6	56.7	42.3
6	—	96.4	0.0	0.0	2.9	—	—	2.9	93.6
7	100.0	99.7	0.0	0.0	3.2	41.1	20.5	54.7	45.2
8	100.0	99.8	0.0	—	2.6	—	—	2.6	97.2
9	100.0	99.3	0.0	0.0	0.5	28.5	49.0	63.8	36.0
10	100.0	99.1	0.0	0.0	1.6	60.4	44.7	78.4	21.4
*Head‐Neck: VeriSoft*									
1	100.0	94.0	0.0	0.0	0.0	0.0	0.0	0.0	94.0
2	99.8	94.0	0.1	3.3	12.0	35.5	22.4	57.4	39.9
3	57.5	—	—	0.0	0.1	—	—	0.0	0.0
4	99.9	96.5	0.0	0.0	10.1	2.6	1.3	13.5	83.4
5	—	92.6	0.0	0.0	0.1	1.6	0.0	1.7	0.0
6	—	98.3	0.0	0.0	0.6	—	—	0.6	0.0
7	100.0	98.9	0.0	0.0	0.5	20.5	17.8	34.9	64.3
8	100.0	99.2	0.0	—	0.3	—	—	0.3	98.9
9	100.0	98.2	0.0	0.0	0.0	49.0	43.7	71.3	28.2
10	100.0	97.7	0.0	0.0	0.3	44.7	35.4	64.4	34.7

HR, high risk; SR, standard risk.

**Table A3:** TCP/NTCP calculation results for lung patients.

Patient	TCP (%)	NTCP (%)			*P*_I_ (%)	*P*_+_ (%)
	PTV	Esophagus	Heart	Lung		
*Lung: Pinnacle*						
1	74.3	—	1.7	—	1.7	73.0
2	84.3	0.0	0.0	0.0	0.0	84.3
3	80.3	—	—	—	0.0	80.3
4	96.9	—	—	—	0.0	96.9
5	40.2	0.0	—	—	0.0	40.2
6	99.1	0.0	0.0	—	0.0	99.1
7	72.2	—	—	—	0.0	72.2
8	77.2	0.6	1.8	17.7	19.6	62.1
9	54.2	—	—	—	0.0	54.2
10	94.9	0.0	—	—	0.0	94.9
*Lung: VeriSoft*						
1	76.5	—	1.8	—	1.8	75.1
2	90.7	0.0	0.0	0.0	0.0	90.7
3	91.1	—	—	—	0.0	91.1
4	96.8	—	—	—	0.0	96.8
5	98.3	0.0	—	—	0.0	98.3
6	97.5	0.0	0.0	—	0.0	97.5
7	76.8	—	—	—	0.0	76.8
8	78.7	0.2	1.3	14.3	15.5	66.5
9	49.0	—	—	—	0.0	49.0
10	93.3	0.0	—	—	0.0	93.3

**Table A4:** TCP/NTCP calculation results for pelvis patients.

Patient	TCP (%)	NTCP (%)					*P*_I_ (%)	*P*_+_ (%)
	PTV	Bladder	Rectum	Sigmoid	Bowel	Penile Bulb		
*Pelvis: Pinnacle*								
1	79.4	0.8	0.9	0.3	—	0.0	1.9	77.9
2	89.0	0.0	2.4	18.0	—	14.9	31.9	60.6
3	82.8	6.3	0.4	0.5	0.5	38.6	70.8	24.2
4	82.8	4.4	4.2	3.3	—	—	11.4	73.4
5	44.8	0.0	0.0	0.0	0.0	0.0	0.0	44.7
6	79.0	1.2	1.9	2.5	—	0.0	5.5	74.6
7	82.5	0.1	0.8	0.1	—	1.0	1.9	81.0
8	82.9	0.0	0.3	0.4	—	0.5	1.3	81.9
9	82.8	0.2	1.9	2.1	0.4	—	41.9	48.1
10	52.2	0.0	0.0	0.0	—	7.2	7.2	48.4
11	82.6	0.0	1.1	0.0	0.0	11.9	12.8	72.0
12	77.0	0.2	1.5	4.0	0.2	—	20.3	61.4
13	82.8	0.0	1.3	1.0	1.6	8.1	155.4	—45.9
14	80.2	—	—	2.2	0.5	—	49.7	40.3
*Pelvis: VeriSoft*								
1	77.8	0.7	0.7	0.2	—	0.0	1.7	76.5
2	86.8	0.0	2.0	0.0	—	10.3	12.2	76.3
3	75.2	3.6	0.5	0.3	0.2	29.4	47.2	39.7
4	82.2	3.2	4.6	3.1	—	—	10.6	73.5
5	37.1	0.0	0.0	0.0	0.0	0.0	0.0	37.1
6	93.1	0.5	1.4	1.7	—	0.0	3.6	89.8
7	80.8	0.1	0.5	0.1	—	0.3	0.9	80.1
8	79.4	0.0	0.2	0.3	—	0.2	0.7	78.9
9	79.6	0.1	2.0	1.9	0.2	—	24.1	60.5
10	47.0	0.0	0.0	—	—	0.0	0.0	47.0
11	78.1	0.0	0.5	0.0	0.0	6.0	6.5	73.0
12	72.9	0.1	1.7	4.3	0.1	—	14.8	62.1
13	79.2	0.0	1.6	0.6	1.1	4.4	110.7	—8.4
14	78.3	—	—	1.6	0.3	—	28.1	56.3

**Table A5:** TCP/NTCP calculation results for prostate patients.

Patient	TCP (%)	NTCP (%)				*P*_I_ (%)	*P*_+_ (%)
	PTV	Bladder	Rectum	Sigmoid	Penile Bulb		
*Prostate: Pinnacle*							
1	95.3	1.4	0.4	0.8	0.5	3.1	92.3
2	97.5	0.6	3.9	21.9	3.1	27.7	70.5
3	96.0	2.7	15.7	34.2	5.7	49.1	48.9
4	98.0	0.2	4.5	0.0	0.0	4.7	93.4
5	97.2	2.5	5.4	—	—	7.7	89.7
6	97.3	3.3	3.4	—	48.9	52.3	46.5
7	97.4	2.9	7.8	12.5	3.9	24.7	73.3
8	96.0	1.7	4.5	0.0	0.0	6.2	90.1
9	99.9	36.9	18.3	36.0	70.0	90.1	9.9
10	97.4	8.2	9.6	26.2	18.5	50.1	48.6
*Prostate: VeriSoft*							
1	92.2	1.0	0.3	1.0	0.2	2.5	89.9
2	95.3	0.3	3.3	18.7	2.8	23.8	72.6
3	93.3	2.0	12.4	30.6	2.7	42.0	54.1
4	94.0	0.0	3.2	0.0	0.0	3.2	91.0
5	93.3	0.5	3.1	—	—	3.6	90.0
6	96.2	2.1	3.3	—	44.5	47.5	50.5
7	95.7	1.3	8.5	9.0	3.2	20.4	76.2
8	86.2	0.4	2.4	0.0	0.0	2.7	83.8
9	99.7	31.4	14.9	31.1	71.3	88.5	11.5
10	95.0	4.3	10.6	23.0	17.2	45.5	51.8
